# Nuclear Factor Y (NF-Y) Modulates Encystation in *Entamoeba* via Stage-Specific Expression of the NF-YB and NF-YC Subunits

**DOI:** 10.1128/mBio.00737-19

**Published:** 2019-06-18

**Authors:** Dipak Manna, Upinder Singh

**Affiliations:** a Division of Infectious Diseases, Stanford University School of Medicine, Stanford, California, USA; b Department of Microbiology and Immunology, Stanford University School of Medicine, Stanford, California, USA; University of California Los Angeles

**Keywords:** *Entamoeba*, developmental biology, encystation, transcription factor, transcriptional regulation

## Abstract

Nuclear factor Y (NF-Y) is a heterotrimeric transcription factor composed of three subunits, namely, NF-YA, NF-YB, and NF-YC, which are conserved throughout evolution. In higher eukaryotes, NF-Y plays important roles in several cellular processes (development, cell cycle regulation, apoptosis, and response to growth, stress, and DNA damage) by controlling gene expression through binding to a CCAAT promoter motif. We demonstrated that NF-Y subunits in the protist *Entamoeba*, while significantly divergent from those of higher eukaryotes, have well-conserved domains important for subunit interactions and DNA binding and that NF-YB and NF-YC are developmentally expressed during encystation. Electrophoretic mobility shift assays confirmed that the NF-Y protein(s) from *Entamoeba* cysts binds to a CCAAT motif. Consistent with a role as a transcription factor, the NF-Y proteins show nuclear localization during development. Additionally, we demonstrated that NF-YC localizes to the chromatoid body (an RNA processing center) during development, indicating that it may have a role in RNA processing. Finally, silencing of the NF-YC subunit resulted in reduced stability of the NF-Y complex and decreased encystation efficiency. We demonstrated that the NF-Y complex functions at a time point subsequent to the NAD^+^ flux and expression of the transcription factor encystation regulatory motif-binding protein, both of which are early regulators of *Entamoeba* development. Taken together, our results demonstrate that the NF-Y complex plays an important role in regulating encystation in *Entamoeba* and add to our understanding of the transcriptional networks and signals that control this essential developmental pathway in an important human pathogen.

## Abstract (precis)

The human parasite Entamoeba histolytica is an important pathogen with significant global impact and is a leading cause of parasitic death in humans. Since only the cyst form can be transmitted, blocking encystation would prevent new infections, making the encystation pathway an attractive target for the development of new drugs. Identification of the genetic signals and transcriptional regulatory networks that control encystation would be an important advance in understanding the developmental cascade. We show that the *Entamoeba* NF-Y complex plays a crucial role in regulating the encystation process in *Entamoeba*.

## INTRODUCTION

Entamoeba histolytica is a protozoan parasite that has caused invasive disease in up to 50 million people worldwide and is a leading parasitic cause of death (1, 2). The most common forms of disease are amebic colitis and dysentery, although infections of the skin, lung, and brain have been reported. *Entamoeba* has two life cycle stages: a cyst form, which can survive in environmental extremes and transmit disease to the next host, and a trophozoite (Troph) form, which migrates into tissue and causes invasive disease. Both life cycle stages are crucial to the organism; however, despite being a central factor in amebic biology, stage interconversion is extremely poorly understood at the molecular level.

The reptilian parasite Entamoeba invadens can be encysted efficiently in the laboratory and is used as a model system to understand the signaling mechanisms by which differentiation (from trophozoites to cysts) is triggered (3, 4). Calorie restriction by glucose starvation and hypo-osmotic shock are the first and foremost environmental factors which trigger the encystation process under laboratory conditions. Two types of receptor-mediated signaling pathways have important roles in encystation: (i) pathways operating through the binding of galactosidase (Gal)-terminated ligands, provided by serum in the media, to *Entamoeba* Gal/Gal-NAc receptors (5, 6); and (ii) adrenergic receptor (AR)-mediated signaling pathways operating through the binding of catecholamine compounds norepinephrine and epinephrine (Epi) (4). Other factors such as cyclic AMP (cAMP) (7), calcium signaling (8, 9), and synthesis of cholesteryl sulfate (10) and phospholipase D (PLD), which are involved in lipid second messenger signaling (11), have important roles in *Entamoeba* encystation. Most recently, the metabolic cofactor NAD^+^ has been shown an important role in encystation (12). Overall, NAD^+^/NADH levels were found to be elevated during encystation and the presence of extracellular NAD^+^ was found to enhance encystation *in vitro* (12).

These observations provide important insights into the conditions that may trigger developmental changes in *Entamoeba*, although the genetic factors regulating these responses are poorly understood. Therefore, identification of the genetic signals and transcriptional regulatory networks that control stage conversion would be an important advance in understanding stage conversion in this parasite. Only a small number of DNA motifs and transcription factors have been characterized in *Entamoeba* (13). *Entamoeba* possess an atypical TATA element (GTATTTAAA) located approximately 30 nucleotides (nt) upstream of the transcription initiation site (14), and E. histolytica putative TATA binding protein (TBP) has significant sequence divergence from the TATA binding protein of Drosophila melanogaster, Caenorhabditis elegans, and Plasmodium falciparum (15). A GAAC element (AATGAACT) and an initiator (Inr) element (AAAAATTCA) overlying the transcription initiation site were also reported earlier as representing a core promoter in *Entamoeba* (16, 17). In *Entamoeba*, transcription factors regulate gene expression relevant to many important aspects of amebic biology, including virulence, oxidative stress response, and stage conversion (12, 18–20).

Some transcription factors have been identified which have important roles in stage conversion of *Entamoeba* (12, 18). A developmentally regulated form of E. histolytica Myb (EhMyb-dr) belonging to the SHAQKY family of Myb proteins binds to a hexanucleotide CCCCCC motif, and overexpression of EhMyb-dr upregulates 117 genes in the encystation pathway (18). Another developmentally regulated transcription factor, encystation regulatory motif-binding protein (ERM-BP), is an NAD^+^-dependent transcription factor which binds the CAACAAA motif and is found in the promoters of 131 cyst-specific genes (12). However, considering that 900 genes are upregulated during encystation, the entire transcriptional network operating during encystation is not well understood and there are likely other factors that regulate encystation.

We analyzed developmentally regulated genes to identify transcription factors which may control stage conversion. Our analysis revealed that the nuclear factor Y (NF-Y) complex (composed of three subunits, NF-YA, NF-YB, and NF-YC) is developmentally regulated in *Entamoeba* (11). Using electrophoretic mobility shift assays (EMSA), we demonstrated that the NF-Y protein(s) from *Entamoeba* cyst nuclear extract binds a CCAAT motif. Silencing of NF-YC resulted in reduced stability of the complex, mislocalization of NF-YA during encystation, and a significant reduction in encystation efficiency. We demonstrated that the NF-Y complex follows ERM-BP and NAD^+^ induction, defining a temporal framework for transcriptional control of *Entamoeba* development.

## RESULTS

### The NF-Y complex was upregulated in amebic cysts.

**FIG 1:**
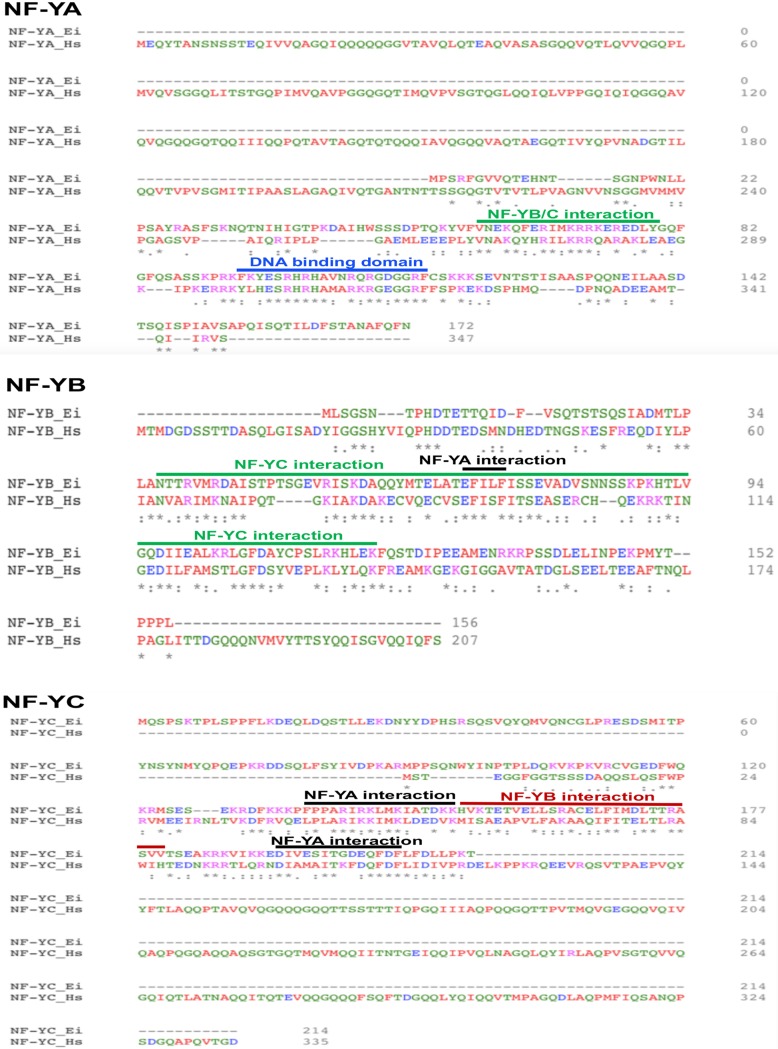
Protein sequence alignment of E. invadens and human NF-Y subunits. Protein sequence alignment of all three NF-Y subunits from E. invadens (*Ei*) and human (*Hs*) was performed by using clustal-omega. Regions identified as required for DNA-binding and subunit interactions of the NF-YA subunit as well as the NF-YB and NF-YC subunits are underlined.

In order to identify transcription factors that may be regulated during development, we searched *Entamoeba* developmentally regulated genes for transcription factors (11, 24). We identified homologues of nuclear factor Y (NF-Y) that are upregulated during E. invadens stage conversion. In eukaryotes, NF-Y is composed of three different subunits, NF-YA, NF-YB, and NF-YC, which form a complex and bind to CCAAT boxes in promoters of target genes (23). We found that the amino acid sequences of all three subunits (EIN_249270 NF-YA, EIN_057000 NF-YB, and EIN_380690 NF-YC) are present in E. invadens and are also well conserved in all *Entamoeba* species that form cysts (see Table S1 in the supplemental material). The *Entamoeba* NF-YA and NF-YC subunits are divergent from homologues in human and other eukaryotes (25, 26); however, the domains essential for NF-Y subunit interactions and DNA binding are conserved in *Entamoeba* (Fig. 1), suggesting that the NF-Y transcription factor complex might be fully functional in *Entamoeba*. Previous work in higher eukaryotes has shown that the N-terminal region of human NF-YA (amino acids [aa] 31 to 140) contains a glutamine-rich domain, which is the minimal domain required for the interaction with Zinc-fingers and homeobox-1 protein (ZHX-1) (27). An amino acid sequence consisting of positions 141 to 269 and containing a serine/threonine-rich domain is the minimal domain required for the interaction with serum-responsive factor (SRF) (27). Amino acid sequence 1 to 212 of NF-YA corresponding to human is missing in *Entamoeba*; thus, the interaction with other proteins through this N-terminal region of NF-YA may not occur in *Entamoeba*.

10.1128/mBio.00737-19.5 TABLE S1 BLAST search of protein sequences of all three NF-Y subunits among different *Entamoeba* species. Results of a BLAST search showing the gene identifier (ID) and maximum (Max) identity and E values for all three NF-Y subunits among E. invadens, E. histolytica, E. dispar, E. moshkovskii, and E. nuttali are listed. Download Table S1, PDF file, 0.04 MB. Copyright © 2019 Manna and Singh. 2019 Manna and Singh This content is distributed under the terms of the Creative Commons Attribution 4.0 International license.

On the other hand, *Entamoeba* NF-YC has a unique N-terminal sequence (89 aa, absent in human) and the C- terminal sequence of NF-YC (aa 121 to 335, corresponding to human NF-YC) is missing in *Entamoeba*. It is reported that the C-terminal sequence of NF-YC interacts with c-Myc in human and that deletion of amino acid sequence 101 to 335 in NF-YC produces a result that mimics an interaction with c-Myc (28). NF-YC is also reported to interact with TBP, and the interacting domains of NF-YC which interact with TBP are conserved in *Entamoeba* (29). The C terminus of NF-YC interacts with other proteins in plant systems. In Arabidopsis thaliana, the QQS protein interacts with the C terminus of NF-YC, plays an important role in the regulation of metabolic processes affecting carbon and nitrogen partitioning among proteins and carbohydrates, and modulates leaf and seed composition (30). Deletion of amino acid sequence 73 to 162 in AtNF-YC4 abolished the binding to QQS (30).

To further define stage-specific expression, we performed semiquantitative reverse transcriptase PCR (RT-PCR) in E. invadens trophozoites and cysts at different time points of encystation (24, 48, and 72 h) (see Fig. S1A in the supplemental material). We found that NF-YA (EIN_249270) was constitutively expressed both in trophozoites and in cyst. However, EIN_057000 (NF-YB) and EIN_380690 (NF-YC) were stage specifically expressed during encystation of *Entamoeba*. NF-YB (EIN_057000) was undetectable in both trophozoites and was upregulated only at later time points (48 h and 72 h) of encystation. EIN_380690 (NF-YC) was undetectable in trophozoites and upregulated early (24 h) during encystation and continued to show high levels at later encystation time points (48 to 72 h) (Fig. S1A and data not shown).

10.1128/mBio.00737-19.1 FIG S1 Cyst-specific expression of NF-Y subunits and CCAAT motif enriched in promoters of genes upregulated during encystation. (A) RT-PCR shows the expression levels of all three NF-Y transcripts in E. invadens (IP1) trophozoites (Trophs) and cysts (72 h) with a loading control (EIN_327460) and an encystation control (EIN_162500). For each sample, reactions were performed with reverse transcriptase (+RT) and without reverse transcriptase (-RT) as a control. (B) The CCAAT motif was found in the promoter of 354 cyst-specific genes and was found throughout 500-nt region upstream of the start codon (ATG). Download FIG S1, PDF file, 0.1 MB. Copyright © 2019 Manna and Singh. 2019 Manna and Singh This content is distributed under the terms of the Creative Commons Attribution 4.0 International license.

### Localization and enrichment of NF-YA and NF-YC in the nuclei of amebic cysts.

In order to analyze the expression of NF-Y subsets at the protein level, we identified commercial polyclonal antibodies to human NF-YA and NF-YC that correspond to regions with high homology to the amebic proteins; a commercial antibody that would recognize the amebic NF-YB was not identified. Western blot analysis performed with human NF-YA antibody in amebic lysates detected a band at below 25 kDa, in both trophozoites and cysts, which was consistent with the predicted molecular mass of *Entamoeba* NF-YA (19 kDa) and consistent with its mRNA expression in both amebic trophozoites and cysts (Fig. S2A). As expected, the NF-YA antibody recognized a protein of around 40 kDa in human cells (Fig. S2A). Cellular fractionation revealed that NF-YA was present at similar levels in nuclear extract (NE) and cytosolic extract (CE) in trophozoites; however, an enrichment of NF-YA in nuclear extract in cysts was observed (data not shown). Western blot analysis performed with human NF-YC antibody resulted in detection of a single band in amebic lysate at ∼25 kDa, which is consistent with the predicted molecular mass of *Entamoeba* NF-YC (23 kDa); the blot demonstrated stage-specific expression of NF-YC in cysts consistent with the earlier mRNA expression data (Fig. S2B). As expected, the NF-YC antibody recognized a protein at 59 kDa in human cells (Fig. S2B).

10.1128/mBio.00737-19.2 FIG S2 Western blot using human NF-YA, NF-YC, and DDX4 antibodies. (A) Whole-cell lysates (40 μg) from E. invadens trophozoites (Trophs), cysts, and human foreskin fibroblast (HFF) whole-cell lysates (HFF) were loaded and probed with anti-NF-YA (sc-17753) antibody. (B) Entamoeba invadens cyst nuclear extracts (NE) and HFF whole-cell lysates were loaded at different concentrations (40 to 80 μg) and probed with anti-NF-YC (ab232909) antibody. (B) Entamoeba invadens cyst nuclear extracts (NE) and HFF whole-cell lysates were loaded at different concentrations (40 to 80 μg) and probed with anti-DDX4 (2F9H5) antibody. Anti-beta actin (ab227387) was used as a control in all experiments. Download FIG S2, PDF file, 0.9 MB. Copyright © 2019 Manna and Singh. 2019 Manna and Singh This content is distributed under the terms of the Creative Commons Attribution 4.0 International license.

**FIG 2:**
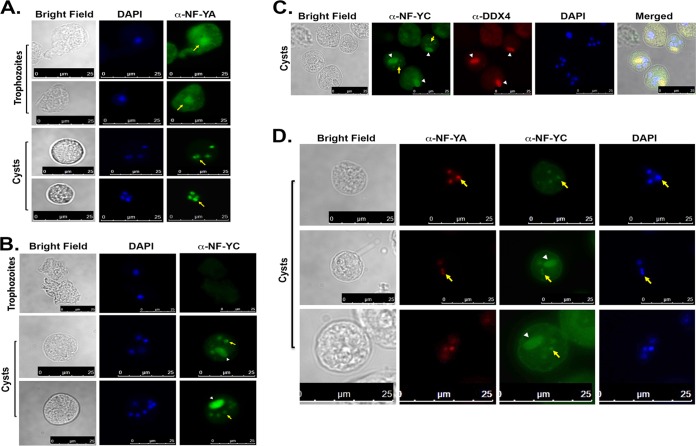
Subcellular localization of NF-YA and NF-YC in *Entamoeba*. (A) Immunostaining with anti-NF-YA antibody in trophozoites (Troph) and 48-h cysts revealed that the protein was expressed in both stages and localized to the nuclei in cysts (green). DNA was stained with DAPI (blue). The yellow arrow indicates nuclear localization. (B) Immunostaining with anti-NF-YC antibody in trophozoites and 48-h cysts (green) shows no signal in trophzoites but localization to the nuclei and chromatoid body in cysts. DNA was stained with DAPI (blue). The white arrowheads indicate chromatoid bodies (CB), and the yellow arrow indicates nuclear localization. (C) Immunostaining with anti-NF-YC (green) and anti-DDX4 (red) was performed in 48-h encysted E. invadens cells. DNA was stained with DAPI (blue). The white arrowheads indicate chromatoid bodies (CB), and the yellow arrows indicate nuclear localization. (D) The 48-h E. invadens cysts were stained with anti-NF-YA (red) and anti-NF-YC (green). DNA was stained with DAPI (blue). The white arrowheads indicate chromatoid bodies (CB), and the yellow arrows indicate nuclear localization. Bars, 25 μm. Both NF-YA and NF-YC localized to the nuclei in cysts, but only NF-YC localized to the chromatoid body.

To determine the subcellular localization, an immunofluorescence assay (IFA) was performed. Immunostaining with NF-YA antibody in E. invadens demonstrated localization in the cytosol and a faint signal in the nucleus of trophozoites (Fig. 2A). In cysts, however, NF-YA localized exclusively to the nucleus (Fig. 2A). Immunostaining with antibody to NF-YC revealed no staining in trophozoites and localization to the nucleus only in cysts (Fig. 2B). In addition to the nuclear localization, we also observed localization of NF-YC in the form of dense patches which resembled chromatoid bodies (CB) within cysts (Fig. 2B; white arrowhead). Chromatoid bodies are rod-shaped or bar-shaped cellular inclusions that have been reported in *Entamoeba* cysts (31). In order to determine if the staining was in chromatoid bodies, we performed coimmunostaining using anti-NF-YC antibody and anti-DEAD box RNA helicase (DDX4) antibody, which was used as a marker for the presence of chromatoid bodies in mouse testes (32). Western blot analysis performed with human anti-DDX4 antibody in amebic lysates detected a band at around 37 kDa and a band at around 75 kDa in human cell lysate, as expected (Fig. S2C). Staining with anti-DDX4 antibody (in red) colocalizes with NF-YC (in green) (shown with white arrowheads in Fig. 2C) provided substantial evidence of localization of NF-YC to the chromatoid body (Fig. 2C). The chromatoid body localization is very specific for NF-YC, however, as NF-YA does not show localization into the chromatoid body (Fig. 2D). Our quantitative analysis in E. invadens cysts identified localization of NF-YC in the nucleus only (63% ± 11%) and in both the chromatoid body and the nucleus (27% ± 8%), as well as in the chromatoid body only (11% ± 3%) (Fig. S3).

10.1128/mBio.00737-19.3 FIG S3 Quantitative analysis of subcellular localization of NF-YC. At 48 h, encysted cells were stained with anti-NF-YC antibody, and percentages of cells with NF-YC nuclear localization only, localization on both the chromatoid body and nucleus, and localization on the chromatoid body only are shown. Data represent means ± SD (*n* = 2). Download FIG S3, PDF file, 0.01 MB. Copyright © 2019 Manna and Singh. 2019 Manna and Singh This content is distributed under the terms of the Creative Commons Attribution 4.0 International license.

### *Entamoeba* cyst nuclear protein(s) bound specifically to the CCAAT motif.

**FIG 3:**
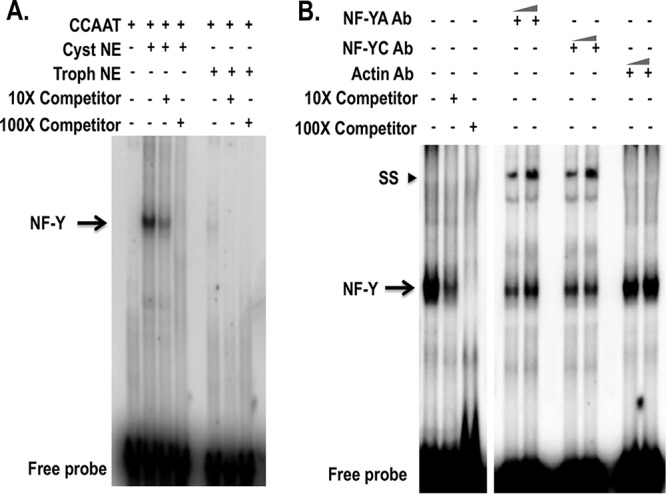
The CCAAT motif specifically bound the NF-Y protein complex in cyst nuclear extract. (A) Representative EMSA results determined in the presence and absence of different components (marked “+” and “−” respectively) are shown. Radiolabeled CCAAT probe was used in each reaction with crude nuclear extracts (NE) from both trophozoites (Trophs) and cysts. Unlabeled CCAAT probe was used as a specific cold competitor at 10× and 100× as indicated. The arrow indicates the major specific band in the gel shift assay; the free probe is indicated at the bottom of the panel. (B) Gel supershift assay using radiolabeled CCAAT and crude nuclear extracts from cysts with anti-NF-YA, anti-NF-YC, and anti-actin antibodies (Ab) at 1 or 2 μg. The arrow indicates a major band corresponding to specific binding to NF-Y. SS, supershifted bands whose presence was due to complex formation with anti-NF-YA and anti-NF-YC antibodies but not with control anti-actin antibodies.

In other systems, the NF-Y complex binds to a CCAAT motif (21, 23). We analyzed the promoter of 900 cyst-specific genes and identified 354 genes with a CCAAT motif in their promoter regions, which is a significant enrichment relative to the number of occurrences of this motif in the entire promoter set of E. invadens (as determined using the hypergeometric distribution) (*P* < 0.0004), implying that this motif may have an important role in encystation. All the cyst-specific genes with a CCAAT motif are listed in Table S2. To determine where the CCAAT motif resides in the promoters, we analyzed its distribution within 500 nucleotides (nt) upstream from the start codon and found that it was distributed throughout the promoter regions but was not enriched in any specific promoter region (Fig. S1B). In order to determine whether the CCAAT motif binds an amebic nuclear protein(s), we performed an EMSA with radiolabeled CCAAT probe. Our analysis demonstrated that the CCAAT motif showed strong and specific binding to a protein(s) from cyst nuclear extracts whereas nuclear extracts from trophozoites showed a very weak band (Fig. 3A). We performed gel supershift (SS) assays using radiolabeled CCAAT probe, crude nuclear extract from cysts, and antibodies to NF-YA and NF-YC, which resulted in the presence of a supershift (SS) band; EMSA performed with the control actin antibody did not result in a supershift (Fig. 3B). Taken together, the data suggest that the NF-Y complex from cyst nuclear extracts binds to the CCAAT motif in *Entamoeba*.

10.1128/mBio.00737-19.6 TABLE S2 List of cyst-specific genes with the CCAAT motif in their promoter regions. A total of 354 genes which upregulated during 24 h of encystation were found to have a NF-Y motif (CCAAT) in the corresponding promoters. Gene ID, product description, protein length, molecular weight, and PFam description are listed. Download Table S2, XLSX file, 0.02 MB. Copyright © 2019 Manna and Singh. 2019 Manna and Singh This content is distributed under the terms of the Creative Commons Attribution 4.0 International license.

### Silencing of NF-YC decreased encystation efficiency and altered nuclear localization of the NF-Y subunits.

**FIG 4:**
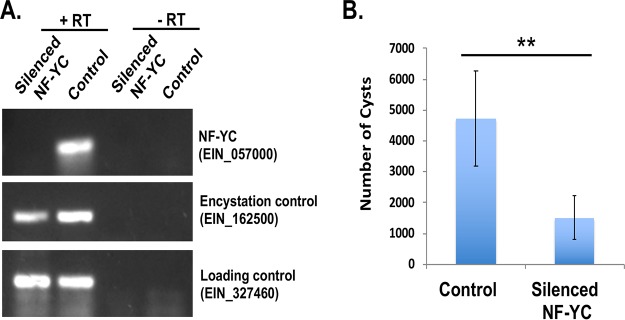
Silencing of NF-YC reduces encystation efficiency. (A) RT-PCR to detect the level of expression of EIN_380690 (NF-YC) transcript in parasites silenced for NF-YC and in control cysts (72 h). A loading control (EIN_327460) and an encystation control (EIN_162500) were included. For each sample, a reaction was performed with reverse transcriptase (+RT) or without reverse transcriptase (-RT) as a control. (B) Data represent the numbers of cysts in control and silenced NF-YC cell lines after 72 h of encystation. The number of cysts in control parasites was compared to the number seen with silenced NF-YC by calcofluor staining, and the data were analyzed by the use of an ImageXpress system (equipped with a laser and image-based acquisition) in a 96-well format. A minimum of 8 wells per parasite line per experiment were analyzed, and biological replicate experiments were performed on three independent days. Data represent means ± standard errors (SE) (*n* = 3) (Student's *t* test; **, *P* < 0.01).

**FIG 5:**
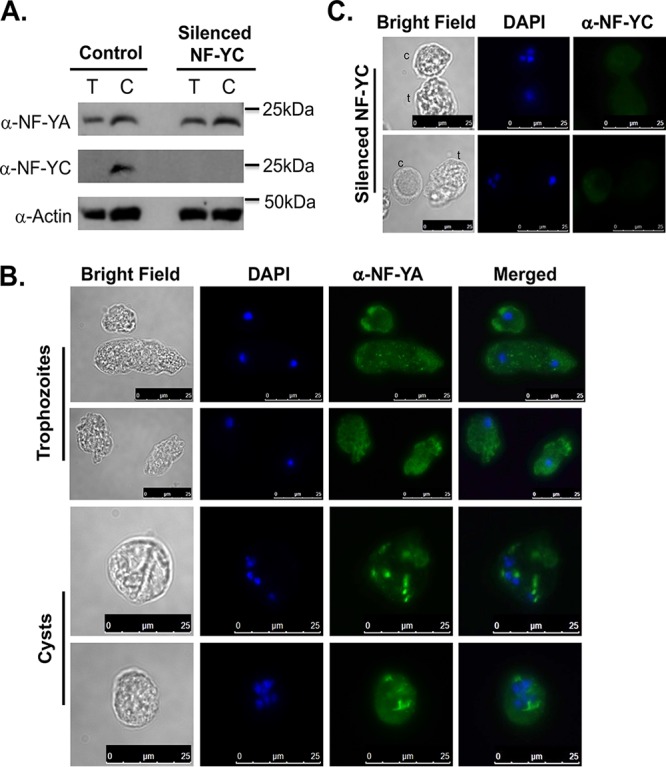
Silencing of NF-YC affects localization of NF-Y. (A) Western blot analysis using anti-NF-YA and anti-NF-YC antibodies in both trophozoites (T) and 48 h cysts (C) in control and silenced NF-YC cells. Silencing of NF-YC did not alter protein levels of NF-YA. Anti-actin antibody was used as a control. (B) Immunostaining with anti-NF-YA antibody in silenced NF-YC trophozoites, and 48 h cyst are shown (green). In NF-YC silenced parasites, localization of NF-YA is altered in trophzoites and cysts. DNA was stained with DAPI (blue). Scale bar for trophozoites and cysts are 25 μm. (C) Results of immunostaining with anti-NF-YC antibody in silenced NF-YC cysts (48 h) are presented (green) and show very low signal levels. DNA was stained with DAPI (blue). Bars, 25 μm.

In order to better understand the role of NF-YC in *Entamoeba* development, we used a trigger-mediated RNA interference gene silencing approach to downregulate NF-YC (33, 34). We were successfully able to silence NF-YC, and the NF-YC transcript level was undetectable in trigger-mediated NF-YC cell lines (Fig. 4A). In order to determine the impact of NF-YC silencing on developmental control, parasites with silenced NF-YC were encysted in 96-well plates and calcofluor-stained cysts were imaged at different time points of encystation (33). Silencing of NF-YC significantly decreased the cyst number at 72 h of encystation, suggesting that the NF-Y complex has an important role in regulating encystation (Fig. 4B). We also calculated encystation efficiency by counting sarkosyl-resistant cysts and demonstrated that the silenced NF-YC parasites showed a significant reduction of encystation efficiency compared to the control (data not shown). Furthermore, we checked the levels of expression of NF-YA and NF-YC protein in parasites silenced for NF-YC. Our Western blot analysis showed no change in the level of NF-YA in trophozoites or cysts in parasites silenced for NF-YC; however, as expected the level of NF-YC protein was undetectable in silenced NF-YC parasites (Fig. 5A). Immunostaining with NF-YA in silenced NF-YC cells showed only cytosolic localization in trophozoites and cysts; the NF-YA localized to punctate focal areas in cysts but did not overlap the parasite nuclei (Fig. 5B). This suggests that although expression of NF-YA does not depend on NF-YC, correct localization into the nucleus during encystation is dependent on the expression of NF-YC. As expected, NF-YC protein was undetectable by IFA in silenced parasites (Fig. 5C).

### NF-Y transcription factor induction followed induction of NAD^+^ and transcription factor ERM-BP.

**FIG 6:**
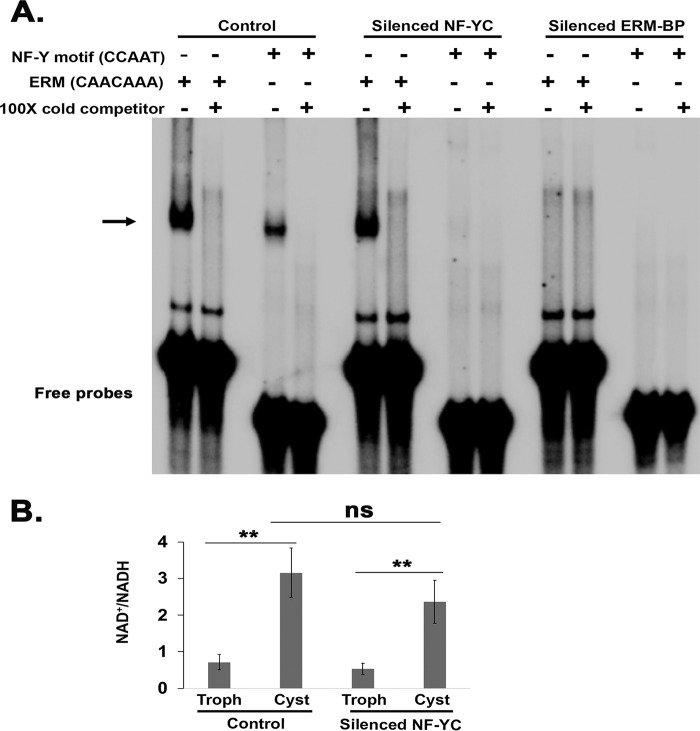
NF-YC induction during encystation temporally follows ERM-BP and NAD^+^/NADH induction. (A) EMSA results are shown in the presence and absence of different components marked “+” and “−,” respectively. Radiolabeled probes for the NF-Y motif (CCAAT probe) and ERM (CAACAA motif) were used as indicated with nuclear extracts from control, silenced NF-YC, and silenced ERM-BP cysts (48 h). Unlabeled probes for each were used at 100× as a specific cold competitor as indicated. The arrow indicates the major specific band in the gel shift assay; the free probes are indicated at the bottom of the panel. In NF-YC-silenced cysts, ERM-BP still functionally bound to its motif. In ERM-BP-silenced parasites, the NF-Y complex did not bind to the CCAAT motif. (B) Measurement of intracellular NAD^+^/NADH in trophozoites (Troph) and 48-h cysts in control and silenced NF-YC parasites. Data represent means ± standard deviations (SD) (*n* = 3) (Student's *t* test; *, *P* < 0.05; ns, not statistically significant).

In order to determine the sequence and appearance of transcription factors during encystation, we next performed EMSA using cyst nuclear extracts from parasites silenced for NF-YC or silenced for ERM-BP and wild-type control cells using radiolabeled NF-Y motif (CCAAT) and the encystation regulatory motif binding protein (CAACAAA motif) (12). As expected, both motifs showed specific binding in cyst nuclear extracts (Fig. 6A). In parasites silenced for NF-YC, the CCAAT (NF-Y motif) binding was largely abolished compared to control cysts; however, binding of the ERM-BP protein CAACAAA motif still occurred. In parasites silenced for ERM-BP, binding of both the ERM-BP and NF-Y protein complexes was significantly reduced (Fig. 6A). We have previously reported that the intracellular NAD^+^/NADH level is elevated during encystation (12). We found that the NAD^+^/NADH level in parasites silenced for NF-YC was increased at 48 h of encystation and was similar to that seen with control parasites (Fig. 6B).

**FIG 7:**
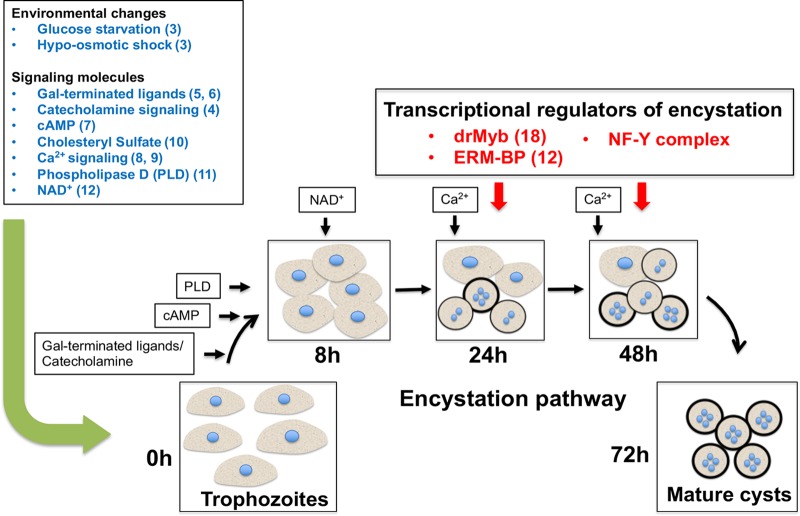
Schematic of regulators of *Entamoeba* encystation. Diverse pathways are implicated in the regulation of *Entamoeba* encystation, where uninucleate trophozoites transformed into quadrinucleate mature cysts. The pathway and the appearance of the key regulators during the different stages of encystation (at 8, 24, 48, and 72 h) are depicted. Environmental factors, including glucose starvation and hypo-osmotic shock, are the key initiators of E. invadens encystation (3). Among the signaling molecules, Gal-terminated ligands (5, 6), catecholamine signaling (4), cyclic AMP (cAMP) (7), cholesteryl sulfate (10), Ca2^+^ signaling (8, 9), phospholipase D (PLD) (11) and NAD (NAD^+^) (12) have shown important roles in encystation. Among the transcriptional regulators of encystation, the roles of two previously described transcription factors (drMyb and ERM-BP) (12, 18) and of nuclear factor Y (NF-Y) (in this present study) and their appearances at different time points of development are illustrated here. References are listed in parentheses in the figure.

Taken together, our data suggest that the pathways involved in expression of ERM-BP and increasing levels of NAD^+^ (12) were not affected in the parasites silenced for NF-YC; thus, the NF-Y function was later than the ERM-BP function in the encystation pathway (Fig. 7). We also observed that the cyst-specific genes with a NF-Y motif were largely distinct from cyst-specific genes with the ERM motif (Fig. S4). A total of 354 genes had the NF-Y motif in their promoter (Table S2), and a total of 131 genes had ERM (12), but there were only 19 genes with both motifs in their promoters (Fig. S4) (Table S4). Further studies are needed to understand the full transcriptional network regulated by NF-Y in *Entamoeba*.

10.1128/mBio.00737-19.4 FIG S4 Overlap of genes possibly regulated by ERM-BP and NF-Y. Venn diagrams of overlapping genes with an encystation regulatory motif (ERM-CAACAAA) and NF-Y motif (CCAAT) in the gene promoter are shown. Download FIG S4, PDF file, 0.02 MB. Copyright © 2019 Manna and Singh. 2019 Manna and Singh This content is distributed under the terms of the Creative Commons Attribution 4.0 International license.

10.1128/mBio.00737-19.8 TABLE S4 List of genes having both a NF-Y motif (CCAAT) and ERM (CAACAAA) in the promoter. A total of 29 genes upregulated during 24 h of encystation had both motifs. Gene ID, product description, protein length, molecular weight, and PFam description are listed. Download Table S4, XLSX file, 0.01 MB. Copyright © 2019 Manna and Singh. 2019 Manna and Singh This content is distributed under the terms of the Creative Commons Attribution 4.0 International license.

## DISCUSSION

In higher eukaryotes, the NF-Y transcription factor acts as an activator or repressor, depending on its interaction with HAT (histone acetyltransferase) or HDAC (histone deacetylase), and plays a critical role in development (35). In *Drosophila*, NF-Y functions as a transcriptional activator in the differentiation of R7 photoreceptor cells during development (23). In the plant Arabidopsis thaliana, transcript analysis revealed that all of the NF-Y genes display differential expression patterns during development and as a response to environmental stimuli (36–38). It has also been reported that the specific expression pattern of each NF-Y protein and interactions among the complementary subunits are important for the specific function of NF-Y complexes in transcriptional control (38). We found that the NF-Y complex is functional in *Entamoeba* cysts, provides important contributions to transcriptional control of stage conversion, and occurs downstream of ERM-BP transcriptional control. Our work begins to develop a framework for the network of transcriptional regulators affecting *Entamoeba* development.

All three NF-Y subunits are well conserved in *Entamoeba*, though the NF-YA and NF-YC subunits are shorter than those in human and other eukaryotes; however, the domains essential for subunit interactions and DNA binding are conserved in *Entamoeba* (25, 26). In *Entamoeba* cysts, NF-YC localizes to the nucleus as well as to a dense rod-like structure, the chromatoid body. In many organisms, including *Drosophila*, germ cells are characterized by the accumulation of dense fibrous material into a cytoplasmic structure called the germplasm or nuage. In mammals, the chromatoid body is suggested to be a counterpart of nuage on the basis of its structural features and protein composition (39). Recent studies identified the CB as RNA-processing bodies in somatic cells (32, 39, 40). Dicer and components of microRNP complexes, including Argonaute proteins (Ago), RNA helicase (VASA homolog, Dead-Box RNA helicase) are highly concentrated into the CB (41). CBs are frequently observed in *Entamoeba* cysts, and may contain various amounts of DNA, RNA, and RNA binding proteins and play a role in cyst wall deposition during *Entamoeba* encystation (42). However, the exact function of chromatoid bodies in *Entamoeba* encystation has remained elusive. The localization of the transcription factor NF-YC in *Entamoeba* chromatoid body links transcriptional control of development to other aspects of cellular RNA control and begins to define the molecular components of the CB.

An earlier study in Entamoeba histolytica revealed that the CCAAT motif can act as *cis*-activator element and control the expression of multidrug-resistant P-glycoprotein gene EhPgp1 (43). Differential DNA-protein complex formation results were seen in the multidrug-resistant clone compared to the drug-sensitive clone involved in the regulation of the EhPgp1 gene expression (43). The nuclear factors that bind to these sites were semipurified by affinity chromatography but were not further characterized (44, 45). Whether the protein complex that binds to the CCAAT motif in multidrug-resistant E. histolytica is the same as that which binds to the CCAAT motif in E. invadens cysts is not clear. However, our efforts using both biochemical (EMSA supershift) and genetic (silencing of NF-Y complex) approaches have definitively demonstrated that the NF-Y complex binds the CCAAT motif in E. invadens and is an important regulator of parasite development.

Silencing of NF-YC significantly reduces the encystation efficiency, directly implicating the NF-Y complex as an important regulator of *Entamoeba* development. However, silencing of NF-YC does not affect either intracellular NAD^+^/NADH or ERM-BP binding, suggesting that NF-Y lies downstream of the ERM-BP and NAD^+^ biosynthesis pathway (Fig. 7). Using our data and published information, we outlined a temporal network of control mechanisms regulating *Entamoeba* development (Fig. 7). Further characterization of NF-Y target genes and interacting protein partners will help define the transcription machinery regulated by transcription factor NF-Y in *Entamoeba*.

## MATERIALS AND METHODS

### Parasite culture, transfection, and induction of stage conversion.

E. invadens (strain IP-1) was axenically maintained (46). To make stable transgenic cell lines, parasites were transfected with plasmid DNA by electroporation (47). Stable cell lines were maintained at a G418 concentration of 80 μg/ml unless otherwise stated. To induce encystation, E. invadens trophozoites were incubated in 47% LYI-LG (supplemented with 7% adult bovine serum) in a 96-well plate (48). Cyst numbers were determined by automated quantitative imaging as described in earlier studies (33, 49). Briefly, calcofluor white, which specifically stains chitin in the cyst wall, was added to wells after 48 h or 72 h of encystation. The calcofluor-stained cysts were imaged at ×10 magnification using ImageXpress Micro (Molecular Devices) and quantified by using MetaXpress analysis software (Molecular Devices). The experiment was repeated at least three times with eight replicates for each sample. Data represent means and standard errors, and the *t* test was performed from a well-distributed data set (24 replicates) of each cell line.

### Western blot analysis.

E. invadens trophozoites or cysts at particular time points (24 h, 48 h, and 72 h) were collected and lysed in lysis buffer. Briefly, the cells were lysed by sonication (1 pulse of 10 s for Trophs and 5 pulses of 10 s each for cysts) in lysis buffer containing protease inhibitors (PIC) (1× PIC, 1 mM leupeptin, 1 mM E-64). Protein lysate was resolved by SDS-PAGE and transferred to polyvinylidene difluoride (PVDF) for immunoblotting. The membranes were blotted with antibodies against mouse monoclonal anti-NF-YA antibody (Santa Cruz; catalog no. sc-17753) (1:1,000), rabbit polyclonal anti-NF-YC antibody (Abcam; catalog no. ab232909) (1:1,000), mouse monoclonal anti-DDX4 antibody (Thermo Fisher; catalog no. 2F9H5) (1:2,000), and rabbit polyclonal anti-beta-actin antibody (Abcam; catalog no. ab227387) (1:10,000). Horseradish peroxidase (HRP)-conjugated secondary antibodies against mouse or rabbit were used at 1:10,000 dilution for 1 h at room temperature and signal detected with ECL^+^ (GE).

### Immunostaining.

E. invadens trophozoites and cysts were fixed with acetone/methanol (1:1) and permeabilized with 0.1% Triton X-100. Cells were incubated with 3% bovine serum albumin (BSA) for blocking followed by mouse monoclonal anti-NF-YA antibody (Santa Cruz; catalog no. sc-17753) (1:200) or anti-NF-YC antibody (Abcam; catalog no. ab232909) from rabbit (1:500) followed by Alexa Fluor 488 anti-mouse or anti-rabbit antibody, respectively (Molecular Probes) (1:2,500). Localization was performed with anti-DDX4 antibody (Thermo Fisher) from mouse (1:200), followed by Alexa Fluor 543-conjugated anti-mouse secondary antibody (Molecular Probes) (1:2,500). Slides were prepared using Vectashield mounting medium with DAPI (4′,6-diamidino-2-phenylindole; Vector Laboratories, Inc.) and visualized using a Leica CTR6000 microscope and a BD CARVII confocal unit. Images were analyzed using Leica LAS-AF software.

### Bioinformatics analysis to identify NF-Y promoter motif.

Upstream promoter regions (500 nt) of 900 cyst-specific genes were analyzed to identify DNA motifs as described earlier (20, 50). In brief, MEME was performed with the command line -dna -mod zoops -minw 5 -maxw 5 -minsites 5 -nmotfs 30. The MAST program was utilized to determine the total number of occurrences of each motif in the promoter sequence databases.

### Electrophoretic mobility shift assays (EMSA).

EMSA was performed as previously described (20). The oligonucleotides used in EMSA are listed in Table S3 in the supplemental material. Each motif had an additional 12 nt at the 5′ end and 8 nt at the 3′ end, which created a 5′ overhang after annealing, and were utilized for radiolabeling using Klenow fragments (50). In brief, complementary overlapping probes were annealed and labeled using [α-^32^P]ATP and Klenow fragments (Invitrogen). The binding reaction mixture consisted of a total volume of 20 μl, which included 2 μl 10× EMSA binding buffer (10 mM Tris-HCl [pH 7.9], 50 mM NaCl, 1 mM EDTA, 3% glycerol, 0.05% milk powder, 0.05 mg of bromophenol blue), 10 μg of nuclear extract form trophozoites or 24-h cysts, 2 μg of poly(dI-dC), and 50 fmol of labeled probe. For gel supershift assays, 10 μg of nuclear extracts was preincubated for 3 h at 4°C in HEPES buffer with 1 and 2 μg of each mouse monoclonal anti-NF-YA antibody (sc-17753), rabbit polyclonal anti-NF-YC antibody (ab232909), and rabbit polyclonal anti-beta-actin antibody (ab227387); followed by 30 min of incubation at room temperature with radiolabeled probe (50 fmol). The binding reaction mixtures were loaded onto a 9% nondenaturing polyacrylamide gel and run for 3 h. The gel was fixed, dried, and exposed to a phosphor screen. Gels were imaged using a Personal Molecular Imager (PMI) system with Quantity One software (Bio-Rad).

10.1128/mBio.00737-19.7 TABLE S3 List of oligonucleotides and primers used in cloning, RT-PCR, and EMSA. Download Table S3, XLSX file, 0.01 MB. Copyright © 2019 Manna and Singh. 2019 Manna and Singh This content is distributed under the terms of the Creative Commons Attribution 4.0 International license.

### RNA extraction and RT-PCR.

Total RNA was extracted from trophozoites and cysts using the TRIzol method (Life Technologies). RNA was subjected to DNase treatment (DNase kit; Invitrogen) and reverse transcribed using oligo(dT) primers (Invitrogen). The resultant cDNA (3 μl) was used in subsequent PCRs (25 μl total volume). The number of PCR cycles was set to 30, and 10 μl of PCR products was run on a 1.5% agarose gel. The negative control (minus reverse transcriptase [RT]) was split away before the addition of Superscript RT (Invitrogen) but was otherwise treated like the other samples. The primers used in RT-PCR are listed in Table S3.

### Plasmid construction.

For gene silencing, the 152-trigger construct was used; a full-length coding region of the NF-YC gene was cloned downstream of the Trigger region at the AvrII and SacII sites as described earlier (33). The primers used in cloning are listed in Table S3. The construct was confirmed by sequencing before transfection into E. invadens.

### Measurement of intracellular NAD^+^/NADH levels.

Intracellular NAD^+^ and NADH levels were determined per the manufacturer's protocol (NAD^+^/NADH assay kit) (Abcam; catalog no. ab65348) and as described earlier (12). Briefly 2 × 10^6^ cells were lysed in NAD^+^/NADH extraction buffer by sonication (five pulses at 15 A for 15 s). The lysate was centrifuged at 14,000 rpm, and the supernatant containing NAD^+^/NADH was filtered through a 10-kDa spin column to get rid of enzymes, which may consume NADH rapidly. To detect the NADH in the sample, a decomposition step was performed by heating the samples at 60°C for 30 min; under such conditions, all the NAD^+^ is decomposed while the NADH is still intact. A 100-μl reaction mixture was prepared for each standard, and samples were processed in duplicate in a clear-bottom 96-well plate. The plate was incubated at room temperature for 5 min to convert NAD to NADH followed by addition of 10 μl NADH developer into each well and was incubated at room temperature for 2 h. Optical density (OD) was measured at 450 nm using a plate reader (BioTek Cytation3).

### Statistical analysis.

Student's *t* test was performed for comparisons of two conditions. A *P* value of <0.05 in each independent experiment was considered significant.
