# Supplementary material for: Nuclear Factor Y (NF-Y) Modulates Encystation in Entamoeba via Stage-Specific Expression of the NF-YB and NF-YC Subunits
Source: mBio. 2019 Jun 18;10(3):e00737-19. doi: 10.1128/mBio.00737-19 (PMC6581852; doi:10.1128/mBio.00737-19)
Supplement: TABLE S1 [file mBio.00737-19-st001.pdf]

Supplemental Table-1.

E. invadens		*E. histolytica*			*E. dispar*			*E. moshkovskii*			*E. nuttali*		
**Protein Name**	**Gene ID**	**Gene ID**	**E-value**	**Max Identity**	**Gene ID**	**E-value**	**Max Identity**	**Gene ID**	**E-value**	**Max Identity**	**Gene ID**	**E-value**	**Max Identity**
NF-YA	EIN_249270	EHI_054140	9.00E-40	58	EDI_297980	6.00E-40	54	EMO_005400	2.00E-53	57	ENU1_197780	2.00E-39	57
NF-YB	EIN_057000	EHI_168220	2.00E-41	53	EDI_252580	6.00E-43	53	EMO_099090	8.00E-61	62	ENU1_092220	0.003	26
NF-YC	EIN_380690	EHI_076830	2.00E-42	42	EDI_349160	7.00E-42	66	EMO_065650	1.00E-57	50	ENU1_018680	2.00E-41	42
