# Supplementary figures and images for: Nuclear Factor Y (NF-Y) Modulates Encystation in Entamoeba via Stage-Specific Expression of the NF-YB and NF-YC Subunits
Source: mBio. 2019 Jun 18;10(3):e00737-19. doi: 10.1128/mBio.00737-19 (PMC6581852; doi:10.1128/mBio.00737-19)

FIG S1.

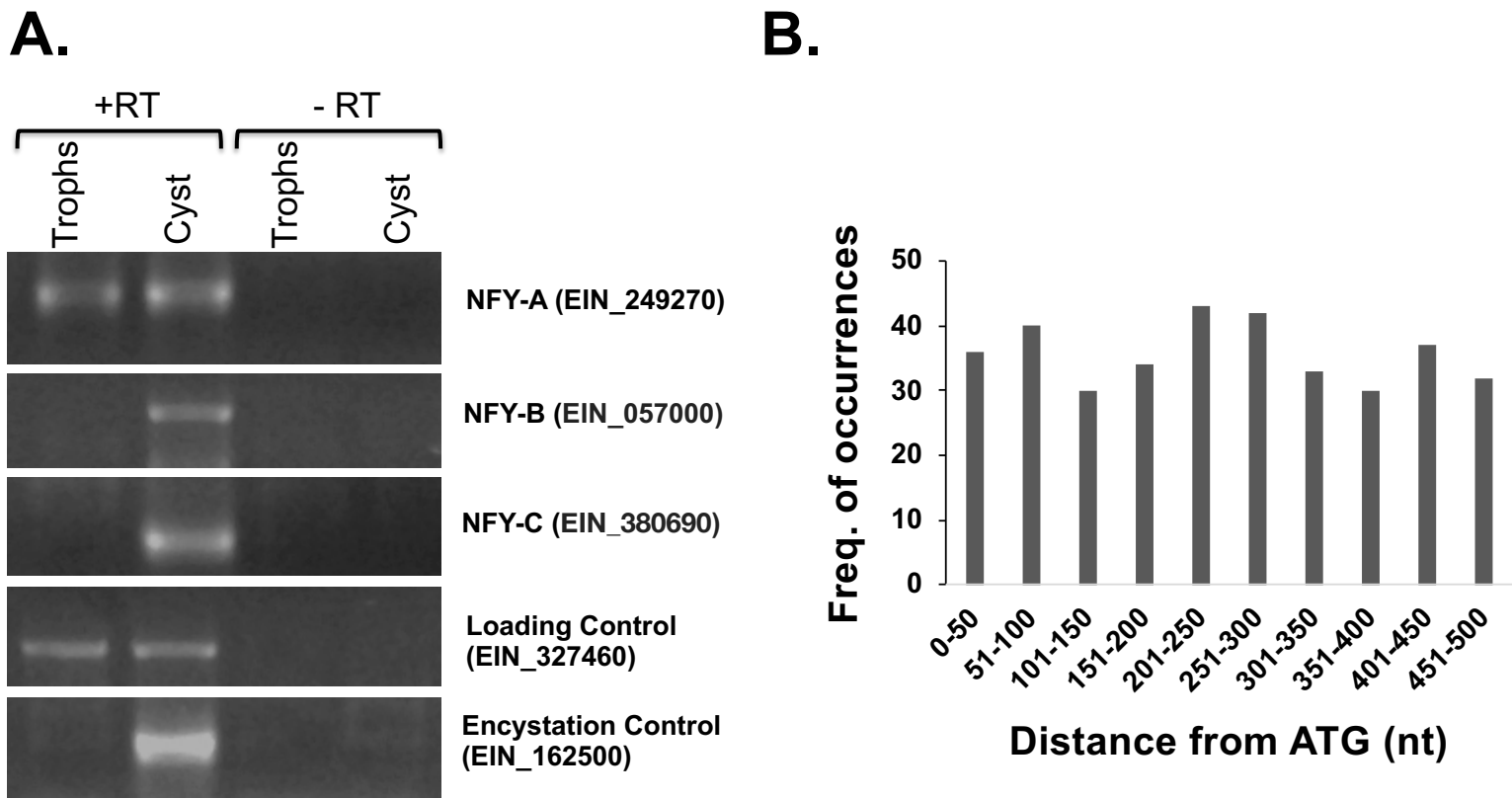

Supplement: FIG S1 [file mBio.00737-19-sf001.pdf]

**FIG S2.**

**A.**

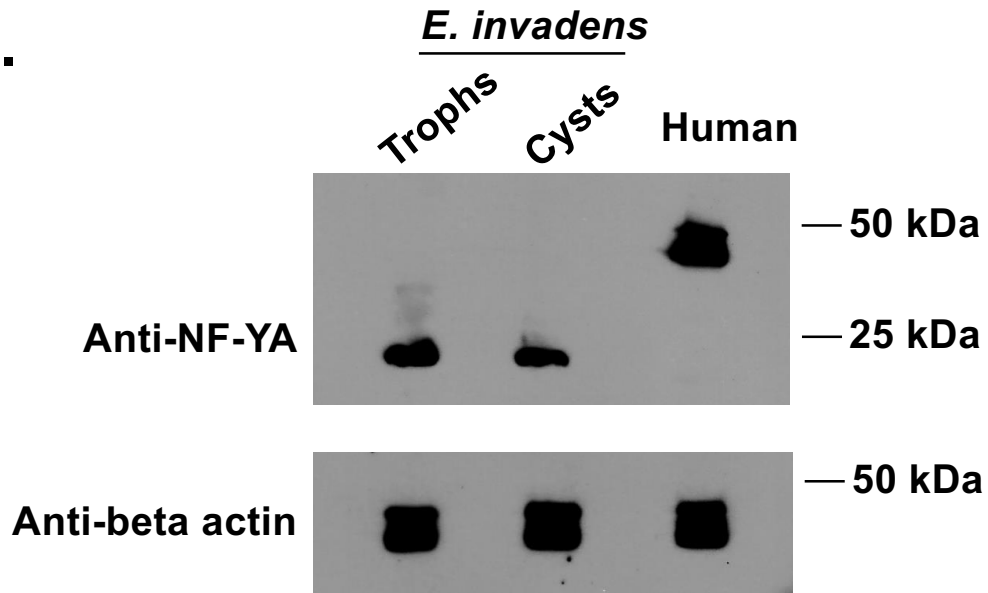

**B.**

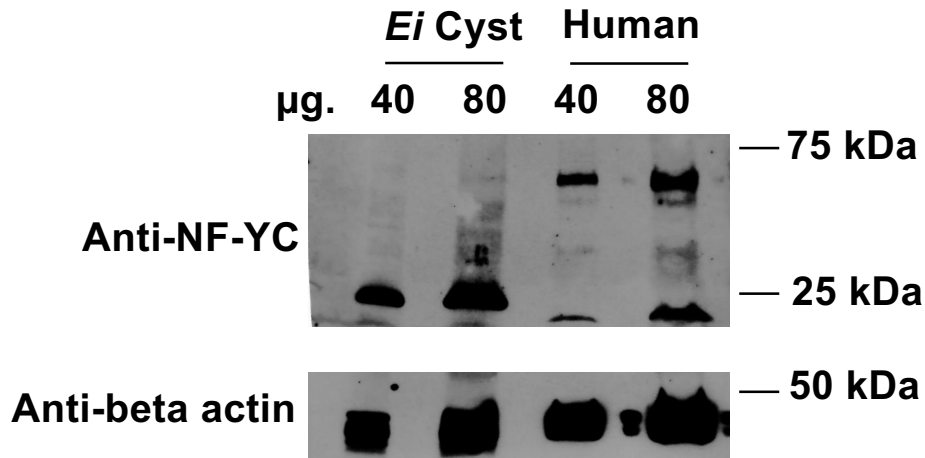

**C.**

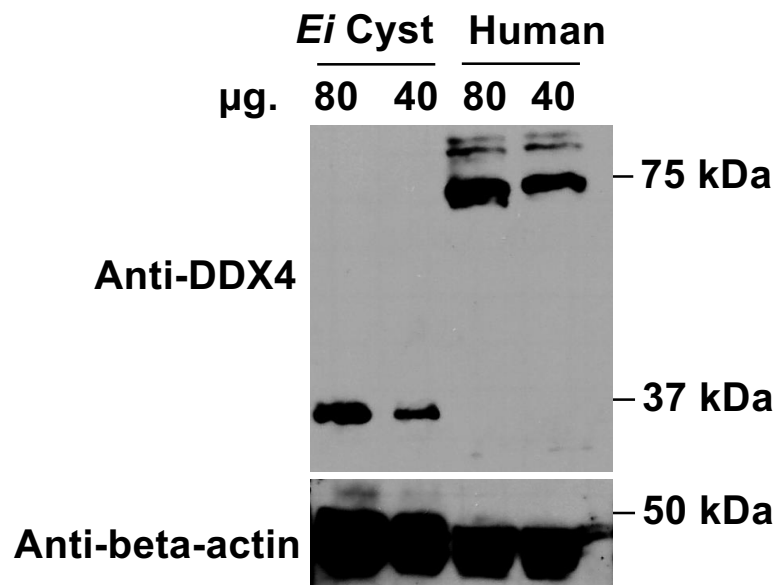

Supplement: FIG S2 [file mBio.00737-19-sf002.pdf]

**FIG S3.**

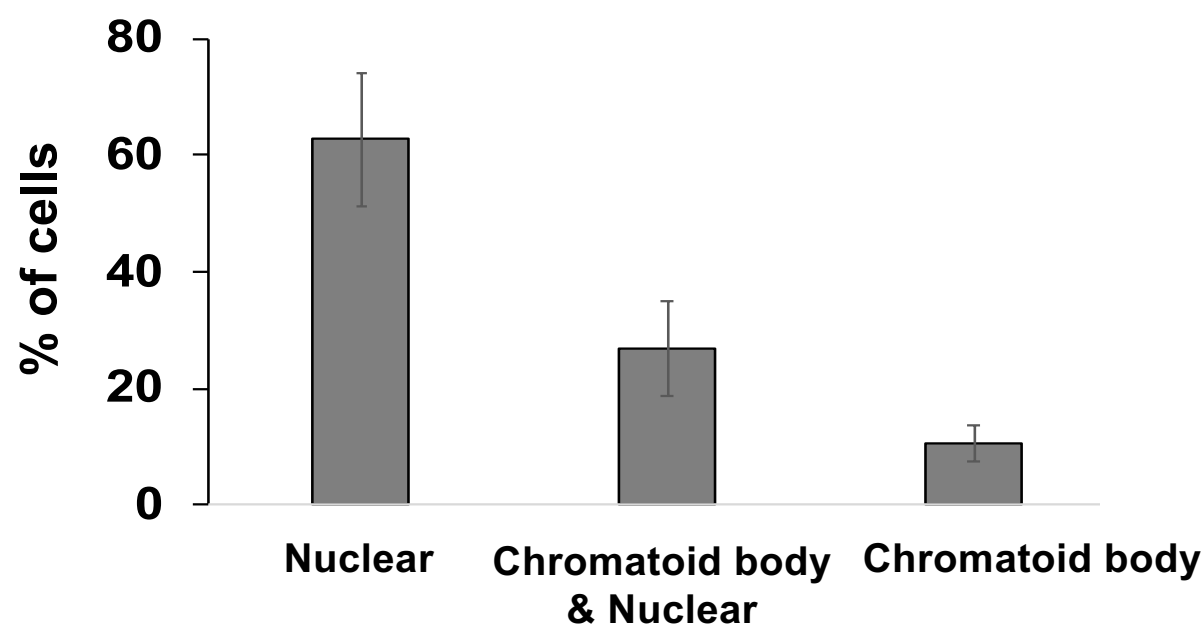

Supplement: FIG S3 [file mBio.00737-19-sf003.pdf]

**FIG S4.**

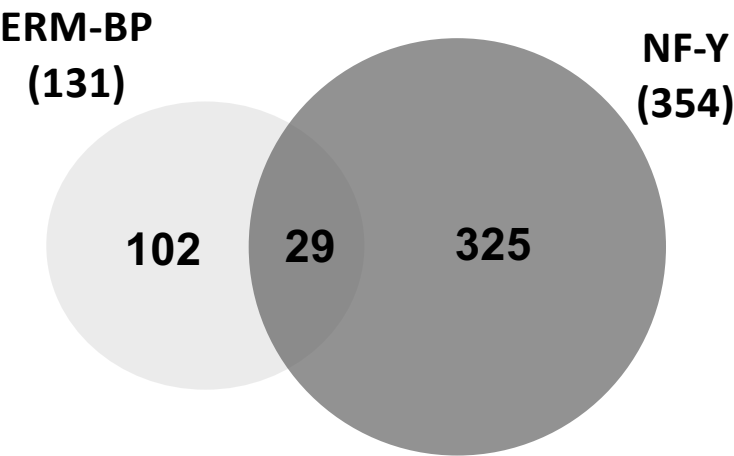

Supplement: FIG S4 [file mBio.00737-19-sf004.pdf]
